# Expression of Two Rye CENH3 Variants and Their Loading into Centromeres

**DOI:** 10.3390/plants10102043

**Published:** 2021-09-28

**Authors:** Elena V. Evtushenko, Evgeny A. Elisafenko, Sima S. Gatzkaya, Veit Schubert, Andreas Houben, Alexander V. Vershinin

**Affiliations:** 1 Institute of Molecular and Cellular Biology, SB RAS, Acad. Lavrentiev Ave. 8/2, 630090 Novosibirsk, Russia; evt@mcb.nsc.ru (E.V.E.); kanopus@ngs.ru (E.A.E.); jait@mail.ru (S.S.G.); 2 Institute of Cytology and Genetics, SB RAS, Acad. Lavrentiev Ave. 10, 630090 Novosibirsk, Russia; 3 Leibniz Institute of Plant Genetics and Crop Plant Research (IPK) Gatersleben, Corrensstr. 3, 06466 Seeland, Germany; schubertv@ipk-gatersleben.de (V.S.); houben@ipk-gatersleben.de (A.H.)

**Keywords:** CENH3 variants, gene duplication, *Secale cereale*, functional DNA motifs, *CENH3* expression, subdomain organization of nucleosomes, CENH3 loading

## Abstract

Gene duplication and the preservation of both copies during evolution is an intriguing evolutionary phenomenon. Their preservation is related to the function they perform. The central component of centromere specification and function is the centromere-specific histone H3 (CENH3). Some cereal species (maize, rice) have one copy of the gene encoding this protein, while some (wheat, barley, rye) have two. Therefore, they represent a good model for a comparative study of the functional activity of the duplicated *CENH3* genes and their protein products. We determined the organization of the *CENH3* locus in rye (*Secale cereale* L.) and identified the functional motifs in the vicinity of the *CENH3* genes. We compared the expression of these genes at different stages of plant development and the loading of their products, the CENH3 proteins, into nucleosomes during mitosis and meiosis. Using extended chromatin fibers, we revealed patterns of loading CENH3 proteinsinto polynucleosomal domains in centromeric chromatin. Our results indicate no sign of neofunctionalization, subfunctionalization or specialization in the gene copies. The influence of negative selection on the coding part of the genes led them to preserve their conserved function. The advantage of having two functional genes appears as the gene-dosage effect.

## 1. Introduction

Gene duplications, which occur as a result of polyploidization following whole-genome duplications (WGDs) or as a result of local duplications of genes and duplication of small genomic regions (small-scale duplications (SSDs)), are thought to have an essential role in generating evolutionary novelty and adaptation [1]. They are common among angiosperms [2], but the mechanisms and evolutionary processes that ultimately determine the fate of duplicated genes are far from being entirely understood. In the simplest case, an SSD gives birth to two copies of a previously single-copy gene. Previous studies identified several possible molecular mechanisms that contribute to the preservation of the second, “daughter” copy in the genome during evolution [3]: (i) *conservation*, in which the function of the ancestral copy is preservedin the daughter due to negative selection aimed at maintaining an increased dose of the gene; (ii) *neofunctionalization*, in which one of the copies acquires a new function as a result of positive selection, while the other retains its ancestral function; (iii) *subfunctionalization*, in which the multiple functions of the parental gene are distributed between two copies [4], is observed either under positive selection, which optimizes the subfunctions of each copy [5], or under neutral selection in the case of unfavorable mutations that weaken subfunctions of each copy [4]; and (iv) *specialization*, in which neofunctionalization is followed by a rapid subfunctionalization, which leads both copies to acquire new functions that are other than the function of the ancestral gene [6]. Subfunctionalization may be accompanied by changes in the subcellular localization of the corresponding protein products [7]. For example, as a result of alternative splicing, the proteins produced by each copy of a duplicated gene can be localized only in one of the cellular structures that they originally occupied (this phenomenon is called *sublocalization*) [7]. 

The first studies seeking to identify centromeric variants of H3 histone (CENH3) in cereals revealed one form of this protein in maize (*Zea mays*) [8] and rice (*Oryza sativa*) [9]. These findings led to the conclusion that the genomes of these species each contain one copy of the corresponding *CENH3* gene. Later studies found that some cereal species in the tribe Triticeae, namely barley, wheat and rye, have two different variants of CENH3 [10,11,12]. The highest heterogeneity was observed in the N-terminal tail (NTT) of CENH3s, which is rapidly evolving to adapt to lineage-specific centromeric constraints [13]. The C-terminus of the CENH3 protein contains a histone fold domain (HFD), which is more conserved and structurally similar to the canonical histone H3. This domain is required for nucleosome assembly and CENH3 targeting to centromeres [14].

The increase in the number of sequenced cereal genomes and advances in their assembly in recent years made it possible to determine the localization of the *CENH3* genes and to trace the evolution of their structure and their surrounding regions in the genomes of various tribes. It was found that around 50MYA, the common ancestor of the subfamilies Bambusoideae, Oryzoideae and Pooideae developed the *CENH3* locus composed of the syntenic genes *CDPK2-l*, *CENH3* and *bZIP*. A duplication event occurred within the *CENH3* locus in Stipeae and Brachypodieae species around 35–40 MYA and produced two genes, *αCENH3* and *βCENH3*. The distance between these genes (the intergenic spacer) is 2.16 kb in *Brachypodium distachyon* and 5.9 kb in *Stipa sibirica* and is filled with relatively short fragments of retrotransposons and tracks of simple repeats [15]. Later in the evolution of the *CENH3* locus in Triticeae, the *CDPK2-l* gene located to the left of *βCENH3* was replaced with the *LHCB-l* gene, which encodes the chlorophyll-binding protein 3C. An increase in the distance between the *βCENH3* and *αCENH3* genes occurred mainly due to massive insertion of the elements of the LTR-containing retrotransposon superfamilies *gypsy* and *copia*. At the same time, the changes in the composition and contribution of individual *gypsy* and *copia* families to the molecular structure of the *CENH3* locus display a significant diversity even in evolutionarily close species [15]. 

The functionality of the *αCENH3* and *βCENH3* genes in Triticeae was studied in the cultivated barley (*Hordeum vulgare*) [16] and diploid and tetraploid wheat species (*Triticum* spp.) [11]. It was demonstrated by immunostaining that in young barley embryos, both variants of the CENH3 protein are loaded into most interphase centromeres. In contrast, in the cells of the root meristem, *βCENH3* is loaded in the centromeres less efficiently than *αCENH3*, and most of the *βCENH3* molecules are dispersed in the nucleoplasm. However, in differentiated tissues, *βCENH3* transcription levels were higher than those of *αCENH3* [16]. Differences in *αCENH3* and *βCENH3* expression were also observed between cultivated and wild wheats [11]. In wild tetraploids, the expression levels of *βCENH3* were significantly lower than those of *αCENH3*; however, in cultivated tetraploids, *βCENH3* transcription levels would increase to nearly reach *αCENH3* levels. 

Similarly to other Triticeae species, two forms of the protein, *αCENH3* and *βCENH3*, were revealed in rye (*Secale cereale* L.) [12]. However, the structure of the entire *CENH3* locus, the expression levels of the genes encoding the CENH3 proteins and the efficiency of these proteins loading into chromosomes remain unknown. The sequencing of two rye accessions [17,18] makes it possible to thoroughly explore the coding and regulatory parts of the *αCENH3* and *βCENH3* genes. In addition, it becomes possible to address how their features correlate with the expression levels of these genes and the localization of the proteins in chromosomes and nuclei at different stages of plant development.In this work, we investigated the organization of the *CENH3* locus in the *S*. *cereale* genome to identify functionally important sites in the vicinity of the *αCENH3* and *βCENH3* genes. The expression levels of these genes at different stages of plant development; the subsequent loading of the products of the expression of these genes, the *αCENH3* and *βCENH3* proteins, into nucleosomes; and the patterns of their organization in linear centromeric chromatin using elongated DNA fibers were analyzed. 

## 2. Results

### 2.1. Molecular Organization of the CENH3 Locus in Rye 

**Figure 1:**
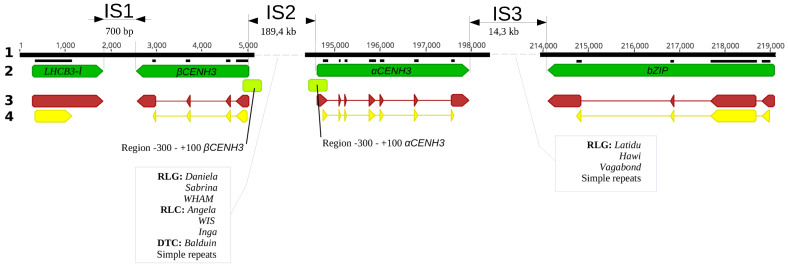
The structure of the *CENH3* locus in rye (*S*. *cereale*). **1:** size in kb; **2:** syntenic genes appear as dark-green fingerpost arrows; **3:** exon–intron structure of genes at mRNA level, where exons appear as fingerpost arrows; **4:** coding regions of genes appear as yellow fingerpost arrows. Light-green rectangles depict −300 and +100 regions around the TSSs of *αCENH3* and *βCENH3*, where functional motifs were searched for.

The availability of the sequenced rye genome (https://www.ncbi.nlm.nih.gov/assembly/GCA_902687465.1, accessed on 9 December 2020) made it possible to describe the structure of the *CENH3* locus and to compare the structure of this locus in closely related species, barley and wheat. The size of the *CENH3* locus in rye line Lo7 [17] from the left-border (upstream) gene, *LHCB3-l* (*LHCB3-like*), which contains the domain of chlorophyll a-b binding protein 3C, to the right-border (downstream) gene, *bZIP*, which is a transcription factor gene, is 218.3 kb (Figure 1). 

The intron–exon structure of the paralogs was determined by analysis of genomic and transcriptome libraries. The *αCENH3* gene has seven exons encoding a protein 166 amino acids in length and is separated by six introns. The *βCENH3* gene has four exons and produces a protein 151 amino acids in length, which is shorter due to an extensive deletion in the NTT domain of the parental gene. Thus, the structure of the *CENH3* locus and the intron–exon structure of the *CENH3* paralogs in rye are similar to those in other Triticeae species [15] but differ from those in *CENH3* paralogs in legumes [19], *Mimulus* [20] and *Arabidopsis* [21]. 

At the *CENH3* locus, the left-border and right-border genes are separated from the *βCENH3* and *αCENH3* genes by intervening spacers. A 189.4 kb intergenic spacer separates *βCENH3* and *αCENH3* (IS2, Figure 1). Most of IS2 is made up of class I transposable elements (TEs) within two retrotransposon superfamilies: *gypsy*-like and *copia*-like, abbreviated *RLG* and *RLC*, respectively, in the notation from unified classification system [22]. Together, their DNA sequences account for 98.2% of all sequences in IS2 identified using the *Viridiplantae RepeatMasker* program. The *RLG-to-RLC* ratio is 2.92, which is only slightly different from the mean value for the rye genome, 3.26. However, the prevalence of separate *RLG* and *RLC* families in IS2 and in the entire rye genome differs more noticeably. For example, the most prevalent families in IS2 (*Daniela, RLG* and *WIS*, *RLC*) are not the most prevalent in rye (*Sabrine, RLG* and *Angela, RLC*) (Table 1). Class I TEs (DTX, transposons) are represented by *Balduin*, a family in the CACTA superfamily, which accounts for only 1.3% of IS2, although the most prevalent DTX family in rye is *Jorge*.

In the 14.3 kb long IS3, *RepeatMasker* identified 79% of DNA sequences, and, with the exception of a very small part composed of simple repeats, the rest of the DNA was found to be made up of three *gypsy*-like families: *Latidu* (56.8%), *Hawi* (36.3%) and *Vagabond* (6.2%). The representation of these families in the entire rye genome is negligible: the respective shares of the most common of them, *Hawi* and *Latidu*, are 1.796 and 1.696 in the *RLG* superfamily. The short IS1, 0.7 kb in length, is devoid of transposon elements, and only one 55 bp sequence appearing as a tandem dimer was identified in it. An exciting feature of the organization of intergenic spacers in the *CENH3* locus is that the coding genes *CENH3* and TEs never occur in adjacency to each other. The insertion sites of TEs begin and end several kilobases away from these genes. These peculiar buffer zones are largely filled with short tracks of micro- and minisatellites and AT-enriched regions. This gradient of different classes of TEs in the vicinity of coding genes is a common rule, at least for Triticeae, because it is characteristic of the entire barley genome [23].

### 2.2. Regulatory Regions of the CENH3 Genes

The observed changes in the structure of the *CENH3* locus and the coding regions of the *αCENH3* and *βCENH3* genes, which took place during the shaping of the rye genome, raise the following questions: To what extent have duplications and related changes affected the structure of the regulatory regions that surround these genes in the course of evolution? How are these regions involved in the regulation of the expression of the *CENH3* genes? Promoters have a complex, gene-specific structure. They may include several short DNA motifs, which serve to bind transcription factors (TFs), and each promoter has a unique composition of these transcription factor binding sites [24]. The core promoter is defined as the shortest DNA motif capable of initiating basal transcription. Typically, it is located from −60 to +40 bp relative to the transcription start site (TSS). The main functions of core promoters are (1) to support the assembly of the preinitiating complex (PIC), which is composed of polymerase II and general transcription factors (GTFs), and (2) to initiate transcription from an exact position and at an exact level [25]. The region from −200 to −300 is normally assigned to the proximal promoter, which contains multiple regulatory elements responsible for the specific regulation of transcription [26]. 

**Figure 2:**
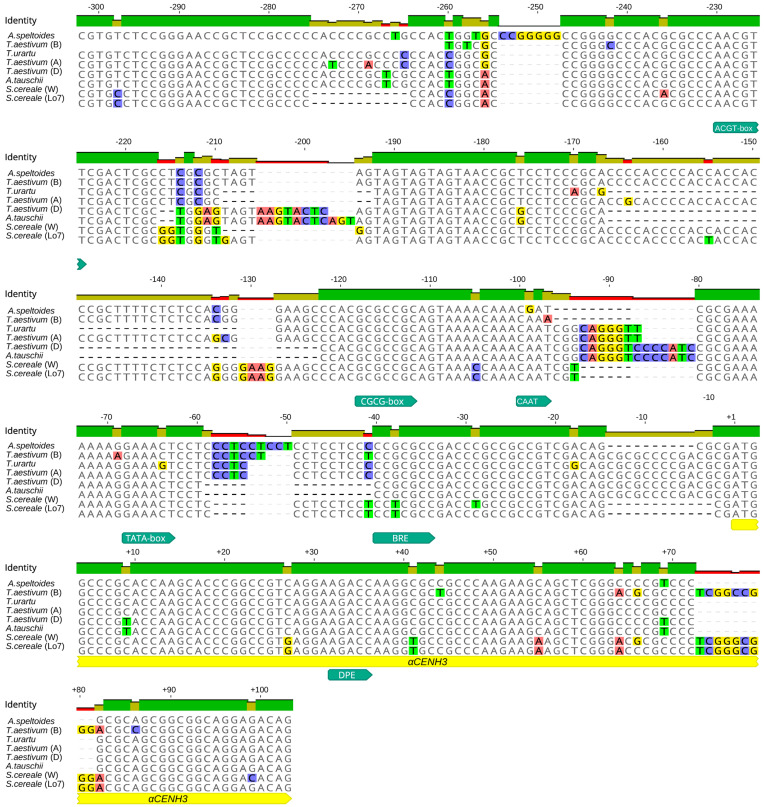
Alignment of nucleotide sequences in −300 and +100 regions around the TSSs of *αCENH3*, where functional motifs were searched for. Functional motifs appear as turquoise fingerpost arrows. The gene transcriptional regions appear as yellow fingerpost arrows.

**Figure 3:**
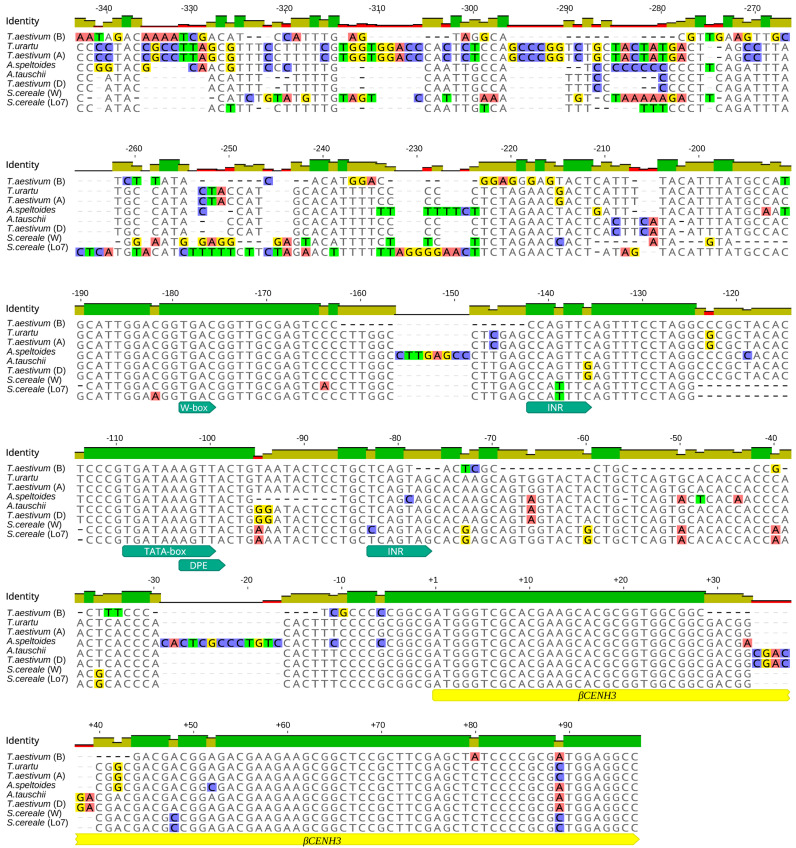
Alignment of nucleotide sequences in −300 and +100 regions around the TSSs of *βCENH3*, where functional motifs were searched for. Functional motifs appear as turquoise fingerpost arrows. The gene transcriptional regions appear as yellow fingerpost arrows.

We analyzed the primary structure of 300 bp regions (−300) upstream of the TSS and 100 bp (+100) downstream of the start of the transcription start sites of *αCENH3* and *βCENH3* (Figure 1). The presence of whole-genome libraries of close-to-rye Triticeae species makes it possible to compare these regions (Figure 2 and Figure 3) in different species using the TSSPlant software program [27]. This program predicts the presence of TATA- containing and TATA-less promoters in the genomic sequences of a wide range of plant species. In all species, six regulatory motifs for the *αCENH3* gene were found in −300 bp regions, including the TATA-box sequence located at position 38 (in rye) from the TSS. The −300 bp regulatory region of the *βCENH3* gene reveals five regulatory elements, among which TSSPlant identifies two pairs of promoters. One pair consists of a TATA box sequence, which is farther from the TSS here than in *αCENH3* (−87 in rye) and presumably functions together with another core promoter, INR (initiator element), located 16 bp downstream of the TATA-box in rye. The TATA-box partially overlaps with the sequence of another downstream promoter element (DPE), which is normally located downstream of the TSS. Presumably, DPE functions in conjunction with another INR located above the TATA-box. Thus, the regulatory region of the *βCENH3* gene has a complex, specific architecture of promoters, given that the promoter elements DPE and INR are characteristic of TATA-less promoters [27]. 

In addition to two pairs of promoter sequences, the regulatory region of the *βCENH3* gene in all Triticeae species contains a TGAC motif (at position −139 in rye), which is known as a W-box or DNA motif for specific binding of WRKY proteins, a TF superfamily [28]. Members of this superfamily participate in the regulation of various plant-specific physiological processes [28]. The TGAC motif is absent in the regulatory region of the *αCENH3* gene, in which, in addition to the TATA-box, five more regulatory elements have been found.The way these regulatory elements are located around *αCENH3* is more consistent with a common pattern than the way they occur around *βCENH3*. For example, the core DPE promoter is located at position +30 downstream of the TSS. In addition to the most common core promoter motifs, TATA and DPE, the *αCENH3* gene contains a recognition element, BRE, to recognize the general transcription factor TFIIB. The entire −300 bp region in the *αCENH3* gene is rich in GC. TSSPlant identifies there a CGCG box upstream of the TATA-box. CG-rich regions serve as a template for epigenetic mechanisms in many promoters and the presence of such promoters is associated with histone modifications [29]. This CG enrichment is characteristic of TATA-less promoters, which suggests that the *αCENH3* gene has in its regulatory region rather a complex architecture consisting of several promoters—as does *βCENH3*. Another functionally important motif in the regulatory region of the *αCENH3* gene is the ACGT box, which is farthest from the TSS (−180 bp in rye). This motif, together with the coupling element (CE), forms part of the ABRC of promoter complexes responding to the action of abscisic acid (ABA), a hormone that inhibits plant growth and development [30]. 

### 2.3. Transcription of the CENH3 Genes

**Figure 4:**
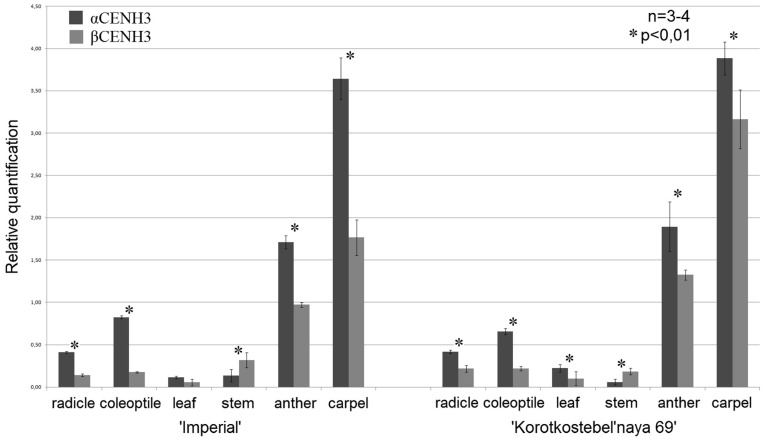
Transcription levels of the *αCENH3* and *βCENH3* genes in different tissues of two rye (*S. cereale*) varieties. Transcription levels normalized to the geometric mean of two references. Each value represents the mean of target/reference ratios with SD bar (linear bar chart). Significant differences between genes for each tissue type are asterisked (*), *p* < 0.01 (Mann–Whitney U test).

The presence of promoters and regulatory motifs around the *CENH3* genes raises the question as to what effect they might have on the behavior of both variants in different tissues during the development of plants. To find out, we compared their transcription levels by qPCR in the rye variety ‘Imperial’ (*S. cereale* subsp. *cereale*) and ‘Korotkostebel’naya69’ (*S. cereale* subsp. *cereale*, K69 throughout) carrying the dominant dwarfing gene *Ddw1*. The results presented in Figure 4 show two general trends. One indicates that the transcription of both variants *CENH3* reaches its highest in the generative tissue. The most remarkable difference is observed between transcription in pistils and transcription in leaves and stems (quantitative data are given in Table S1). It is about 30-fold for *αCENH3* and *βCENH3* in ‘Imperial’ and about 60-fold for *αCENH3* and 30-fold for *βCENH3* in ‘K69’. The other suggests (with one exception) that in both varieties, transcription levels of *αCENH3* are higher than those of *βCENH3* at all developmental stages studied except in stems (Figure 4). The largest difference between the variants is observed in coleoptiles: 4.7-fold in ‘Imperial’ (Table S1) and 3.0-fold in ‘K69’. However, in stems, where transcription is most downregulated, the transcription level of *βCENH3* is 2.4 times higher in ‘Imperial’ and 3.2 times higher in ‘K69’. Thus, it should be noted that both tendencies are characteristic of both varieties, which differ significantly in some phenotypic traits. 

### 2.4. Subdomain Organization of Nucleosomes Containing Different CENH3 Variants in Centromeric Chromatin

**Figure 5:**
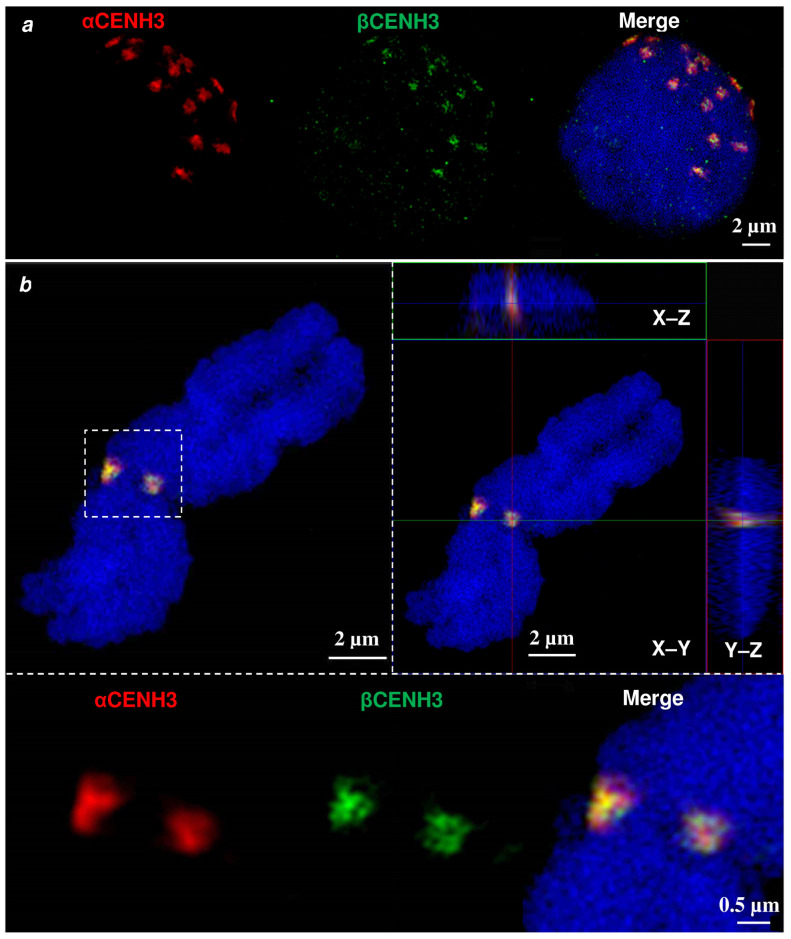
*αCENH3* and *βCENH3* localization in the centromeres of interphase nuclei (**a**) and metaphase chromosomes (**b**) of rye. The increased resolution achieved via SIM reveals that both CENH3 variants colocalize and intermingle. (**a**) The centromere concentration at the top-right clearly visualizes the Rabl orientation in the nucleus. (**b**) The orthoview (right) indicates the spatial distribution of CENH3 within the centromere. Below, the centromeric region (dashed rectangle) is shown enlarged.

To find out whether proteins are synthesized from the *αCENH3* and *βCENH3* variants, whether these proteins are loaded into nucleosomes and how they are organized within the centromeric chromatin, we employed CENH3 variant-specific antibodies in combination with super-resolution spatial structured illumination microscopy (3D-SIM) and confocal microscopy. That the number of immunostaining signals in flow-sorted interphase nuclei isolated from rye seedlings (Figure 5a) was equal to the chromosome number indicates that both *αCENH3* and *βCENH3* are indeed loaded into rye centromeres. The colocalized CENH3 ultrastructures visualized by SIM are intermingled (Figure 5b, Video S1).

Thus, it is likely that rye centromeres contain subdomains of CENH3-free nucleosomes. Analysis of the *αCENH3* and *βCENH3* signals at individual stages of mitosis (Figure S1) demonstrated their presence on chromosomes throughout the entire process of cell division. This suggests a synchronized synthesis and dynamics of incorporation of both variants into nucleosomes. 

**Figure 6:**
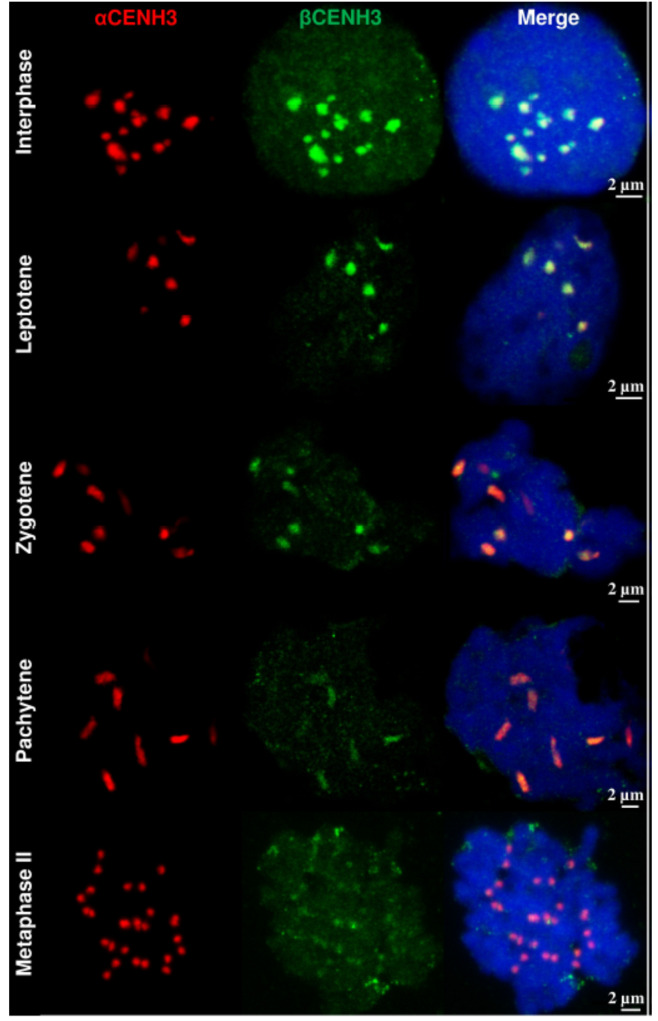
Loading the *αCENH3* and *βCENH3* proteins into rye chromosomes in meiosis.CENH3 variant-specific antibodies in combination with laser confocal scanning microscopy were used for analysis of meiotic stages in young anthers.

A similar trend was observed in the analysis of the dynamics of the *αCENH3* and *βCENH3* signals when young anthers are progressing through meiosis. Figure 6 shows that chromosomes tend to cluster during meiosis. In interphase I of meiosis, CENH3 signals varying in size—3–4 large, composed of associated centromeres, and 3–5 singletons—are located on one side of the nucleus. Putting chromosomes together into a small number of groups at the nuclear periphery is a commonly observed phenomenon, which is centromere coupling involving nonhomologous, pairwise associations [31]. In the zygotene stage, green signals (*βCENH3*) slightly outnumber red signals (*αCENH3*), while in pachytene, when the chromosomes appear more extended, red signals begin to prevail. Because the quantitative fluctuations in the intensity of signals at this level of resolution depend on many factors, and so it is not feasible to assess their influence, the best we can do is confirm that the dynamics of the loading of both CENH3 variants into meiotic chromosomes corresponds to that observed for mitotic chromosomes. 

**Figure 7:**
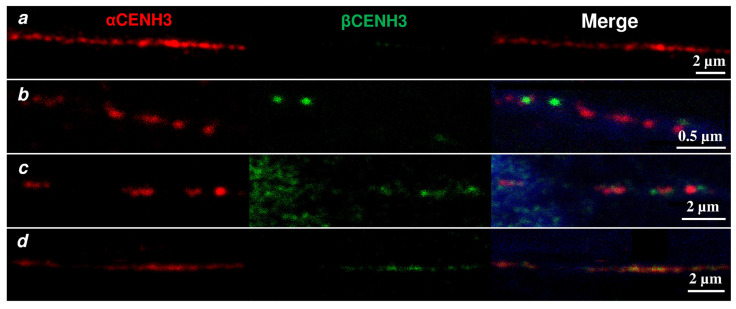
Different types of nucleosome clusters visible on extended chromatin fibers. (**a**) Clusters containing nucleosomes with *αCENH3*-only signals. (**b**) Clusters containing nucleosomes with *βCENH3*-only signals. (**c**,**d**) Examples of clusters containing nucleosomes with *αCENH3* and *βCENH3* signals colocalized. The colocalization values are: **c** = *βCENH3*/*αCENH3*—25–75%, *αCENH3*/*βCENH3*—25–35%; **d** = *βCENH3*/*αCENH3*—56–90%, *αCENH3*/*βCENH3*—22–62%. Clusters with CENH3 signals are interrupted by gaps with nucleosomes containing the canonical histone H3.

To understand the subcentromeric organization of both CENH3 proteins, we used extended chromatin fibers prepared from interphase nuclei isolated from 5-day-old seedlings. The fibers were immunolabeled and analyzed by confocal laser scanning microscopy. Confocal image stacks collected from immunostained clusters for the *αCENH3* and *βCENH3* proteins with different lengths were measured in nanometers and converted to units of length for DNA, a diameter of 11 nm per nucleosome [32] and a mean nucleosome size of 180 bp [33].We measured and quantitatively analyzed the following: (1) the size of clusters consisting only of *αCENH3* signals (red) (Figure 7a), (2) the size of clusters consisting only of *βCENH3* signals (green) (Figure 7b), (3) clusters consisting of alternating signals of both proteins (red, green and yellow signals) (Figure 7c,d) and (4) the sizes of the gaps between the signal tracks. Such gaps were found between the clusters of *αCENH3* signals and tracks consisting of signals of both proteins, with *αCENH3* signals prevailing. They are likely represented by regions of centromeric chromatin, where nucleosomes include the canonical histone H3. 

Table 2 shows the results of the analysis, which reveals two clear-cut tendencies: 

(1) *βCENH3* signals form the shortest clusters, the maximum size of which is about 12 kb, while the maximum size of the clusters of *αCENH3*-only signals reaches about 40 kb.

(2) The greatest range of values is observed in the clusters consisting of signals from both *αCENH3* and *βCENH3* proteins (red, green and yellow) and is from about 5 to 120 kb. We think that such clusters are indeed formed by chromatin regions consisting of alternating nucleosomes with *αCENH3* and *βCENH3*, which is confirmed when using the “Colocalization” option of the software ZEN (Zeiss). Quantitative estimates of colocalization are given in the Figure 7 caption. As the size distribution plots show (Figure S2), the increase in the length of the clusters occurs gradually. Most of them are relatively small in size. The majority of clusters with *αCENH3* signals are found within 1000 nm or approximately 15–16 kb, and most of the clusters with alternating signals of both proteins as well as gaps in signals are found within 1500 nm or 25 kb. A smooth appearance of the plots is disrupted by a sharp increase in the size of the clusters with the signals of both proteins within 2000–7000 nm (or 30–120 kb) in a small number of measurements made (six). Even though the sizes of the extended chromatin fibers and, consequently, the number of clusters with signals can be influenced by various factors, including mechanical ones, the large number of measurements that we have performed allows us to claim that the tendencies noted above reflect the likely picture of the nucleosomal organization of centromeric chromatin in rye.

## 3. Discussion

Gene duplication and the preservation of both copies, parental and daughter, during evolution is an intriguing evolutionary phenomenon, especially if we look at closely related taxonomic entities (tribes, genera and even species) that have been getting by with a single copy for millions of years. Although an important role is attributed to gene duplications in generating evolutionary novelty and adaptation [2], this role is often not so evident. The four possible molecular mechanisms supporting the preservation of the daughter copy in the genome during evolution [3] are not equally widespread across taxa. A comparison of the expression profiles between the ancestral single-copy gene in one species and the parent and child copies arising from an SSD event in a closely related sister species demonstrated that 65% of duplicated genes in Drosophila have undergone *neofunctionalization* [34]. By contrast, the use of this approach with eight mammalian species suggests that only 33% of the duplicated genes have been preserved by *neofunctionalization*, while the rest (i.e., most) carry on due to the *conservation* of functions [35]. This tendency appears to be also characteristic of plant species, which is confirmed by an impressive analysis performed by Jiang and Assis [3]. From three cereal species, the authors formed 272 SSD-derived gene pairs in *Brachypodium distachyon*, 289 pairs in *Oryza sativa japonica* and 340 pairs in *Sorghum bicolor*. It was demonstrated that duplicated genes in all these species traverse similar evolutionary paths after SSD and 60.6% of these duplicated genes were performing the same function as the ancestral copy (that is, they were conservative), 23.8% had acquired a new function (that is, they became *neofunctionalized*), 0.4% had become *subfunctionalized* and 15.2% had become specialized [3]. Thus, *conservation* is the most preferred mechanism with these species; it preserves the duplicated copy in the genome during evolution, which leads to an increase in gene dosage [35]. Interestingly, the *CENH3* locus consisting of the syntenic genes *LHCB3-l, CENH3* and *bZIP* is also present in *B*. *distachyon* and *O*. *sativa* [15]. However, in *O*. *sativa*, the *CENH3* gene occurs in the locus as a single copy, while in *B*. *distachyon*, it occurs as two copies—just as in rye (Figure 1)—strongly suggesting that whether or not a particular gene would become duplicated could rather be a chance circumstance.

No matter what molecular mechanism of duplication, DNA-mediated or RNA-mediated, the copies find themselves in a new genomic context outside their ancestral regulatory elements, hence the expectation that the copies will become neofunctional or subfunctional. That is why it was somewhat surprising that, according to the above publications, the copies do not live up to expectations. A comparison of the molecular compositions of DNA sequences in the *CENH3* locus between rye and Stipeae and Brachypodieae species that are the first cereals to have evolved two variants of the *CENH3* gene (about 35–40 MYA) shows a general tendency towards the expansion of the *CENH3* locus. An increase in the size of the locus and, consequently, in the distance between *βCENH3* and *αCENH3* genes was due to the massive introduction of various families of TEs, mainly of the main LTR-containing retrotransposon superfamilies, *gypsy* and *copia.* A comparison of the composition and prevalence of these families between this locus and the genomes of different Triticeae species revealed substantial differences [15]. In this respect, the *CENH3* locus in rye is different from the rest of the genome too (Table 1). However, hardly could these differences exert any effect on the expression levels of the *βCENH3* and *αCENH3* genes: the insertion sites of TEs begin and end several kilobases away from the genes, and these peculiar buffer zones are largely filled with short tracks of micro- and minisatellites, AT-enriched regions and unidentified sequences. 

The alignment of the primary structure of −300 and +100 regions relative to the TSS, that is, the regions where TF-binding DNA motifs [24] and regulatory elements responsible for specific transcription regulation [25] normally reside, showed that they are highly conserved in both the *αCENH3* and *βCENH3* genes of different Triticeae species (Figure 2 and Figure 3). However, a comparison of these regions with their counterparts in *Brachypodium* species, *B*. *sylvaticum* and *B*. *distachyon*, revealed a very high level of heterogeneity, which prevented their alignment with Triticeae species. Thus, the amount of pressure exerted by negative selection on these regions during the evolution of the *CENH3* locus was rather low. This statement is supported by a comparison of the sets of functional sites between *αCENH3* and *βCENH3*. These sets contain both common regulatory motifs and those specific for each gene. A common feature of both genes is the complex architecture of their promoters; along with TATA boxes identifiable by TSSPlant [27], it contained the DPE and INR motifs characteristic of TATA-less promoters. It is possible that the extensive sets of functional sites make the regulatory regions of the *αCENH3* and *βCENH3* genes so universal. Comparing the expression levels of both genes between different developmental stages of two phenotypically different rye varieties, ‘Imperial’ and ‘K69’, revealed a clear-cut correlation between expression levels and cell division intensity (Figure 4 and Table S1). The transcription level of *αCENH3* prevailed over that of *βCENH3* in all studied stages of plant development, except in stems. The importance of a higher level of *αCENH3* expression is confirmed by the result of an aminoacid exchange leading to impaired centromere loading of *βCENH3*, which, however, did not cause phenotypic changes in barley [36].

Cell division in stems is lowest, and the expression of cell cycle-related genes probably reaches its minimum basal level. This phase of plant development was the only one at which the transcription level of the *βCENH3* gene was higher than that of *αCENH3*. This fluctuation might be due to the presence of the functionally important motif ACGT box in the regulatory region of the *αCENH3* gene. This motif is part of ABRC promoter complexes responding to the action of abscisic acid (ABA), a hormone that inhibits plant growth and development [30]. Because this motif is not found in the vicinity of the *βCENH3* gene, the inhibitory effect of the hormone is probably exerted on *αCENH3* to a higher degree. Together with our comparison of the transcription levels of the *αCENH3* and *βCENH3* genes at different stages of plant development, the presence of the proteins synthesized from these transcripts in chromosomes at different stages of mitosis and meiosis reveals no sign of neofunctionalization, subfunctionalization or specialization in the copies. The influence of negative selection (purifying selective pressure) on the coding part of the *βCENH3* gene [15] led this gene to preserve its conserved function, and the benefit of the presence of two genes normally appears as the gene-dosage effect. Another advantage could be associated with the fact that each copy encodes the N-terminal tail with big differences in amino acid sequences, which is the case for the *αCENH3* and *βCENH3* proteins in rye [12], and may form the basis for different protein–protein interactions [37]. It is possible that new approaches to studying and assessing functional divergence will help reveal the above functions in cereals—in addition to conservation. The study of centromeric functions of the CENH3 variants in cowpea through the CRISPR/Cas9-based inactivation of one of the variants [38] is an example of an informative approach. 

The question of the linear organization of nucleosomes in centromeric chromatin in species expressing more than one variant of the centromere histone H3 is a special question. Assuming that separate clusters of signals correspond to particular nucleosomal subdomains, we identified four main patterns of the co-organization of the CENH3 variants, which correspond to the main polynucleosomal subdomains in centromeric chromatin. It should be noted that not all CENH3 is loaded into chromatin in many species. For example, about 66% of CENH3 was found to be loaded into chromatin outside centromeres in human retinal pigment epithelium cells [39] and about 30% in chicken cells [40]. Not aware of any similar quantitative assessments in plants, we propose that the four patterns of polynucleosomal CENH3 subdomains form part of centromeres. A similar subdomain organization of centromeric chromatin with alternating nucleosomal clusters containing both CENH3 variants and nucleosomes containing the canonical histone H3 was described for centromeres in barley [16], legumes [38,41], humans and Drosophila [42,43]. Thus, the organization of chromatin with polynucleosomal subdomains containing a unique set of histone variants is evolutionarily conserved for centromeres and makes centromeric chromatin different from the neighboring noncentromeric regions [41,42,43]. A high diversity of the sizes of the four patterns suggests that each chromosome of rye has, apparently, a specific organization of polynucleosomal subdomains. This diversity reveals several most extended clusters largely containing signals from both CENH3 variants likely interrupted by small nucleosomal subdomains with the canonical H3 (Figure S2). We propose that such extended subdomains may appear to be a centromeric core surrounded peripherally by shorter subdomains with diverse combinations of histone variants and post-translational histone modifications. 

## 4. Materials and Methods

### 4.1. Identification of the CENH3 Locus in the Rye Genome; Analysis of Its DNA Sequences

The rye genome assembly was downloaded from GenBank (https://www.ncbi.nlm.nih.gov/assembly/GCA_902687465.1, accessed on 9 December 2020). The search for *CENH3* sequences in the genome was performed using the *TBLASTN* program from the AB-BLAST package [44]. The amino acid sequence of rye *CENH3* obtained by us earlier (*ACC AUN88458*) was used as a query [15]. The composition of repeated DNA sequences in the *CENH3* locus was determined using the RepeatMasker version open-4.0.5, sensitive mode, and the *Viridiplantae* database (RepeatMasker-RepBase Sequence Database RELEASE 20170127) [45]. Data were visualized using the *Geneious* 11.0.2 software (http://www.geneious.com, accessed on 9 December 2020) [46]. For comparative analysis, the genomic sequences of *CENH3* in other *Triticeae* species obtained from previously sequenced genomes were used [15]. 

### 4.2. Identification of Functional Sites in the Vicinity of the *αCENH3 and βCENH3*

Multiple alignment of regulatory regions in the vicinity of the *αCENH3* and *βCENH3* genes was performed using MUSCLE, v3.8.31 [47]. Identification of functional sites was performed using TSSPlant and NSITE-PL [48,49] on http://www.softberry.com, accessed on 9 December 2020. Core promoter elements were identified using the online tool YAPP Eukaryotic Core Promoter Predictor (http://www.bioinformatics.org/yapp/cgi-bin/yapp.cgi, accessed on 16 December 2020).

### 4.3. Plant Material

Transcription levels of *αCENH3* and *βCENH3* were determined in vernalized rye plants of two cultivars, *S. cereale* subsp. *cereale,* cv. ‘Imperial’ and *S. cereale* subsp. *cereale,* cv. ‘Korotkostebel’naya69’. The seeds were obtained from the gene banks of the N.l. Vavilov Institute of Plant Genetic Resources, St.Petersburg, Russia (‘Imperial’, accession No. 9368, K69 accession No. 10892), and Leibniz Institute of Plant Genetics and Crop Plant Research (IPK), Gatersleben, Germany (‘Imperial’, accession No. R1771). Plants were grown in a greenhouse (a 16/8 h day/night cycle at a 25 °C/20 °C day/night temperature). The growth stages at which the samples were collected for analysis were characterized by the Zadoks scale (Zadoks 2-digit code [50]): (1) primary roots, 2–3 cm (Z06); (2) coleoptiles, 1.5–2 cm (Z09); (3) leaves at tillering (Z21-Z24); (4) stems at the 2-node stage (Z32); (5) anthers at the stage between meiosis and development of mature pollen (Z42-44); (6) nonpollinated pistils (Z50-52). Tissues of the same type from five different plants were combined into one biological sample.

### 4.4. RNA Extraction and cDNA Synthesis; RT-qPCR

Total RNA was extracted from the biological samples using the TRIzol method (TRI Reagent, MRC, Inc., OH, USA) and treated with DNase using a DNA-*free* DNA Removal Kit (Invitrogen, Thermo Fisher Scientific, Waltham, MA, USA). First-strand cDNA was synthesized using FireScript Reverse Transcriptase (R04-50, BiolabMix, Novosibirsk, Russia) in a volume of 20 μL from 3.6 μg of DNase-treated total RNAEach PCR reaction was carried out in a volume of 25 μL and contained 12.5 μL BioMaster HS-qPCR SYBR Blue (2×) (BiolabMix, Novosibirsk, Russia), 8 μL of first-strand cDNA diluted 25 times (which corresponds to 0.32 μL of the initial cDNA per reaction) and 0.3 mM of forward and reverse primers. The optimum concentration and temperature for primer annealing had preliminarily been set for each target. Reactions were performed in duplicate for the reference genes and in triplicate for *αCENH3* and *βCENH3* using a LightCycler 480 Instrument II (Roche, Basel, Switzerland) according to the following program: at 95 °C for 5 min, followed by 45 cycles at 95 °C for 15 s, at the annealing temperature of 62 °C for 20 s, and at 72 °C for 30 s. 

Because the literature had nothing about reference genes suitable for comparative transcription analysis in the rye genome, the reference genes used for the normalization of the transcription levels of the rye genes *αCENH3* and *βCENH3* were Ta2776 (RNase L inhibitor-like protein) and Ta53967 (Vacuolar ATP synthase 16 kDa proteolipid subunit), selected following the search for the best reference genes for various tissues of *T*. *aestivum* [51]. For amplification of the transcripts of the rye reference genes, primers were selected taking into account the differences between wheat and rye sequences of the reference genes, for which purpose we used contigs of *S*. *cereale* orthologous genes (BioSample: SAMEA3928734BioProject: PRJEB13501, https://www.ncbi.nlm.nih.gov/biosample/SAMEA3928734/, accessed on 20 September 2020). Primers used in the study are listed in Table S2. The amplification products of the reference genes were checked by electrophoresis and were found to be consistent with the expected lengths of the amplicons.

The primers for amplification of the *αCENH3* and *βCENH3* transcripts (Table S2) were designed using an IDT PrimerQuestTool (https://eu.idtdna.com/pages/tools/primerquest/, accessed on 18 November 2020) at the exon boundaries in order to exclude the influence of genomic DNA [12]. Consistency between the reaction products and expected products was checked by sequencing. The relative quantification was carried out using the standard curve method [52]. External standard curves for each gene were constructed in a series of six five-time dilutions. The results obtained were analyzed using the LC480 software (section 1.5.1.62) supplied with the LightCycler 480. The primer efficiency ranged from 96.6% for *βCENH3* to 102.6% for *αCENH3* and 100% for the reference genes. The comparison of the transcription levels of *αCENH3* and *βCENH3* was performed using the “Relative quantification analysis with high confidence 2nd derivative max method” (LC480 software 1.5.1.62). 

### 4.5. Synthesis of Antibodies

The following peptides were used to obtain polyclonal antibodies: rye *αCENH3*, KKLGTRPSGGTQRRQDTDGAGTSATPRRAGR, in rabbits;rye *βCENH3*, TATTPEKKKRLRFELSPRWR, in mice. Life-Tein (LLC, NJ, USA, www.lifetein.com, accessed on 20 March 2017) and Biosan Company (Novosibirsk, Russia, https://biosan-nsk.ru, accessed on 12 March 2019) performed the peptide synthesis, immunization of rabbits and mice and peptide affinity purification of antisera.

### 4.6. Slide Preparation and Indirect Immunostaining

Seedlings of the rye variety ‘Imperial’ with about 2 cm long main roots were incubated for 20 h at 4 °C in ice water. The nuclei were sorted according to [53]. Mitotic preparations were made from root meristems fixed in Tris buffer containing freshly prepared 3% paraformaldehyde (PFA) under vacuum at 4 °C for 5 min followed by a 20 min fixation at 4 °C without vacuum [54]. After washing for 5 min three times in ice-cold Tris buffer, the meristems of about 20 root tips were chopped up with a sharp razor blade in 1 mL of ice-cold LB01 lysis buffer composed of 15 mM Tris-HC1, 80 mM KC1, 20 mM NaCl, 2 mM disodium EDTA, 0.5 mM spermine, 0.1% (*v*/*v*) Triton X-100 and 15 mM mercaptoethanol, pH 7.5 [55]. This suspension of released chromosomes and nuclei was filtered through a 50 μm pore size filter of Cell Strainer cap (Corning Inc., NY, USA) and kept on ice. Filtrated suspension was diluted 1:10 in LB01 lysis buffer, and 100 µL was centrifuged onto slides by using a Shandon Cytospin 4 (Thermo Fisher Scientific, Waltham, MA, USA) at 2000 rpm for 10 min. The loaded slides were treated with 0.5% Triton X-100 for 5 min at 4 °C, fixed in 4% PFA for 10 min at room temperature (RT) and rinsed for 5 min three times in 1× phosphate-buffered saline (PBS) [56]. Extended chromatin fibers from young etiolated leaf nuclei were prepared as described by Houben et al. [57]. For the analysis of pollen mother cells, meiotic chromosome slides were prepared according to Hesse et al. [58]. 

Immunostaining was performed according to Jasencakova et al. [54] with minor modifications. The nuclei were postfixed in 4% (w/v) PFA in PBS for 10 min, washed three times in PBS and blocked for 1 h at RT in 2xSSC containing 3% BSA and 0.1% Triton X-100. Immunostaining was performed at 4 ⁰C for 48 h with primary antibodies diluted in AK buffer (2xSSC containing 1% BSA and 0.1% Triton X-100). Slides were washed for 5 min three times in 2xSSC, and secondary antibodies diluted in AK buffer were applied for 1 h at 37 °C. Slides were washed for 5 min three times in 2xSSC and afterward mounted and counterstained with 4′,6-diamidine-2′-phenylindole dihydrochloride (DAPI, 1 mg/mL) in Vectashield (Vector Laboratories Inc., Burlingame, CA, USA). The following primary antibodies were used: rabbit rye anti-*αCENH3* (diluted 1:500) and mouse rye anti-*βCENH3* (diluted 1:75). As secondary antibodies, a goat anti-rabbit rhodamine (1:200; Jackson Immuno Research Laboratories, West Grove, PA, USA) and goat anti-mouse Alexa 488 (1:400; Invitrogen, Thermo Fisher Scientific, Waltham, MA, USA) were used. 

### 4.7. Microscopy

To analyze the ultrastructure of FISH signals and chromatin beyond the classical Abbe/Raleigh limit, we applied spatial structured illumination microscopy (3D-SIM) at alateral resolution of ~120 nm (super-resolution, achieved with a 488 nm laser), using a 63×/1.4 oil plan-apochromat objective of an Elyra PS.1 microscope system with the software ZEN Black (Carl Zeiss GmbH, Jena, Germany). Images were captured separately for each fluorochrome using the 405, 488 and 561 nm laser lines for excitation and appropriate emission filters [59]. A SIM image stacks were used to produce the 3D movie with the Imaris 8.0 (Bitplane AG, Zurich, Switzerland) software. Maximum intensity projections were calculated via the ZENBlack software. The male meiosis and extended chromatin fiber images were captured by a confocal laser scanning microscope with a 100×/1.46 oil plan-apochromat objective (LSM 780, Carl Zeiss GmbH, Jena, Germany), with the multitrack configuration for detecting rhodamine (excitation at 561 nm, emission collected by a bandpass of 566–685 nm) and Alexa 488 (excitation at 488 nm, emission collected by a bandpass of 497–558 nm). Laser intensity and gain were set at similar levels for all experiments. Maximum intensity projections were done with the ZEN2010B software. Measurements and colocalization analysis were done by the Line measurement tool and Colocalization function of the ZEN2010B software.

## Figures and Tables

**Table 1:** Contribution of the most abundant class I and class II families to the IS2 and genome of rye.

*Gypsy*-Like (*RLG*) *						*Copia*-Like (*RLC*) *					
Family	% in IS2	% in Superfamily (in IS2)	Family	% in Superfamily (in Genome)	% in Genome	Family	% in IS2	% in Superfamily (in IS2)	Family	% in Superfamily (in Genome)	% in Genome
*Daniela*	22.5	32.3	*Sabrine*	18.2	8.8	*WIS*	10.4	44.8	*Angela*	37.38	5.37
*Sabrine*	16.5	24.5	*Daniela*	10.3	4.9	*Inga*	6.2	26.8	*WIS*	22.65	3.25
*WHAM*	9.3	13.8	*Erika*	6.2	3.0	*Angela*	5.1	22.1	*Barbara*	14.41	2.07
*Erika*	5.3	7.9	*Laura*	6.0	2.9				*Inga*	5.15	0.74
*Romani*	5.2	7.7	*Sabine*	5.8	2.8				*Eugene*	2.79	0.40

* In brackets: codes in accordance with the classification by Wicker et al. [22].

**Table 2:** Characteristics of chromatin fibers with CENH3 variants in the rye centromeric chromatin.

Types of Clusters with Variants of CENH3	Clusters Measured	Size, nm	Size, kb *
Clusters containing nucleosomes with *αCENH3*-only signals	97	278–2588	4.5–42.3
Clusters containing nucleosomes with *βCENH3*-only signals	27	202–714	3.3–11.7
Clusters containing nucleosomes with *αCENH3* and *βCENH3* signals colocalized	48	293–7205	4.8–117.9
Size of gaps between clusters with CENH3 signals	72	206–5399	3.4–88.3

* To calculate cluster sizes in kilobases, we assumed that the chromatin fibers have approximately the same and evenly distributed degree of stretching. The diameter was set at 11 nm per nucleosome [31], and the mean size of nucleosomes was set at 180 bp [32].

## Supplementary Materials

The following are available on line at https://www.mdpi.com/article/10.3390/plants10102043/s1, Figure S1: Loading the *αCENH3* and *βCENH3* proteins into rye chromosomes at different mitotic stages. CENH3 variant-specific antibodies in combination with laser confocal scanning microscopy were used for analysis of mitotic rye cells at early prophase, late prophase, metaphases, anaphaseand telophase (half of the telophase is shown). Figure S2: Size distribution of different types of nucleosome clusters in centromeric chromatin in rye (data from Table 2). On the x-axis: number of measurements made; on the y-axis: size of clusters in nm. Red curve: distribution of clusters with *αCENH3* signals; green curve: distribution of clusters with *βCENH3*; yellow curve: distribution of clusters with *αCENH3* and *βCENH3* signals; dark-blue curve: distribution of gaps between clusters with signals telling that the nucleosomes contain the canonical histone H3. Table S1: Normalized transcription levels of *αCENH3* and *βCENH3* in different tissues of rye (*S*. *cereale*). Table S2: List of primers used in this study. Video S1: A spinning rye metaphase chromosome demonstrating the loading and centromeric localization of *αCENH3* (red) and *βCENH3* (green).
