# Supplementary material for: Expression of Two Rye CENH3 Variants and Their Loading into Centromeres
Source: Plants (Basel). 2021 Sep 28;10(10):2043. doi: 10.3390/plants10102043 (PMC8538535; doi:10.3390/plants10102043)
Supplement: Supplementary file 1 [file plants-10-02043-s001.zip › Figure S1.pdf]

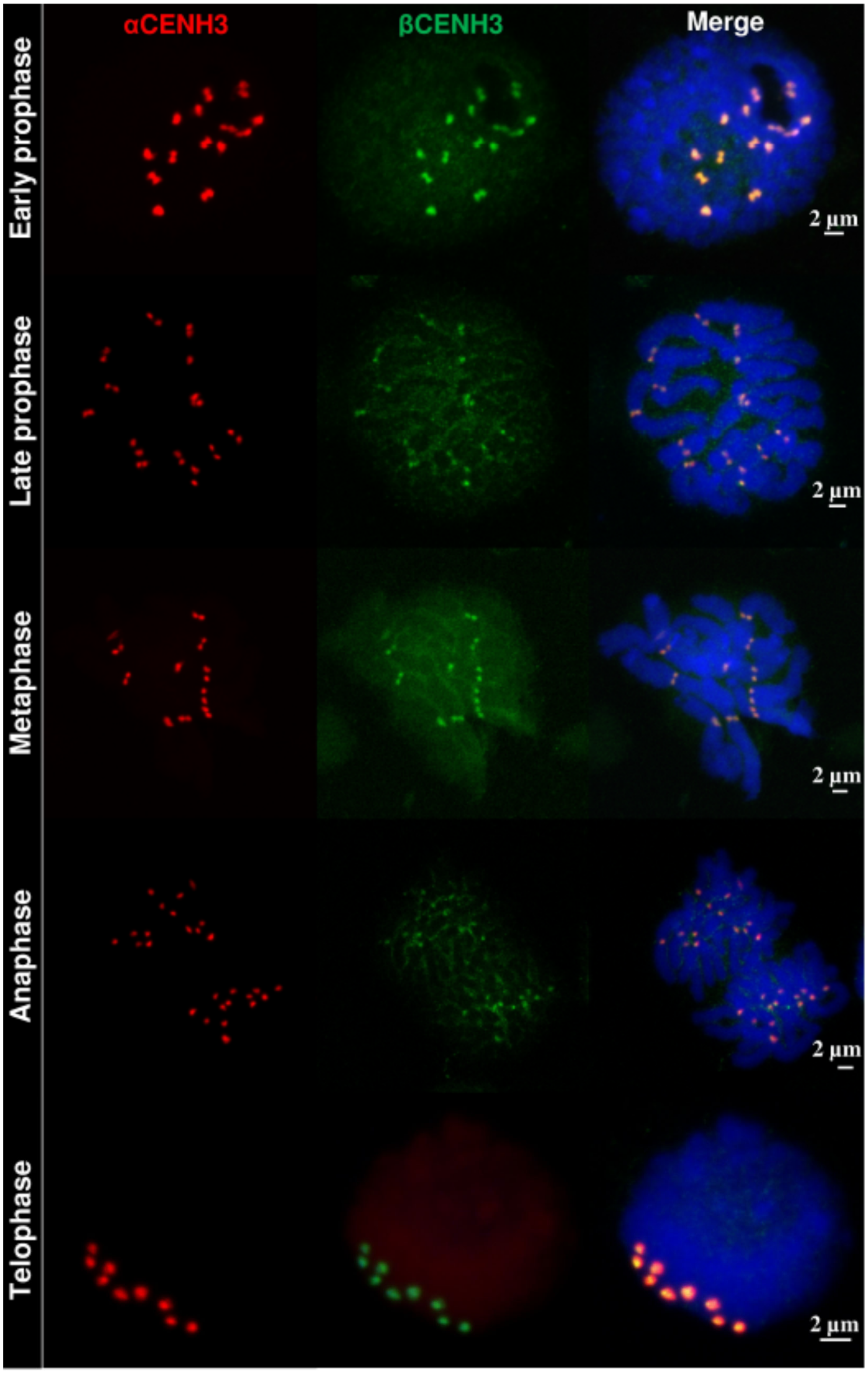

Figure S1. Loading the αCENH3 and βCENH3 proteins into rye chromosomes at different mitotic stages.

CENH3 variant-specific antibodies in combination with laser confocal scanning microscopy were used for analysis of mitotic rye cells at early prophase, late prophase, metaphases, anaphase and telophase (half of the telophase is shown).
