# Supplementary material for: Expression of Two Rye CENH3 Variants and Their Loading into Centromeres
Source: Plants (Basel). 2021 Sep 28;10(10):2043. doi: 10.3390/plants10102043 (PMC8538535; doi:10.3390/plants10102043)
Supplement: Supplementary file 1 [file plants-10-02043-s001.zip › Figure S2.pdf]

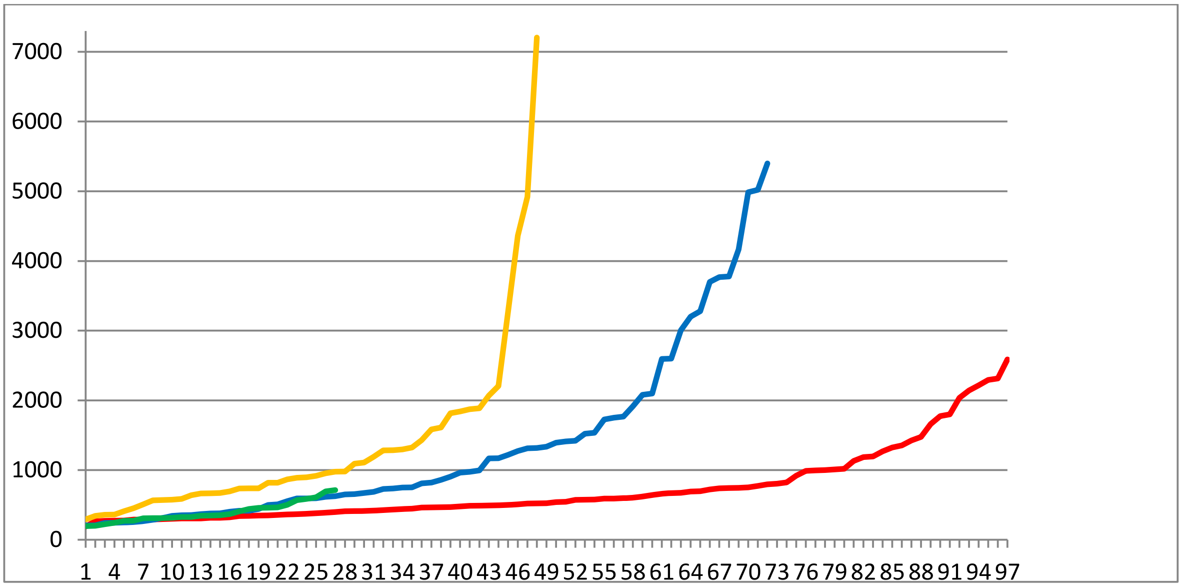

Figure S2. Size distribution of different types of nucleosome clusters in centromeric chromatin in rye (data from **Table 2**)

On the *x*-axis: number of measurements made; on the *y*-axis: size of clusters in nm. Red curve: distribution of clusters with αCENH3 signals; green curve: distribution of clusters with βCENH3; yellow curve: distribution of clusters with αCENH3 and βCENH3 signals; dark-blue curve: distribution of gaps between clusters with signals telling that the nucleosomes contain the canonical histone H3.
