# Supplementary material for: Expression of Two Rye CENH3 Variants and Their Loading into Centromeres
Source: Plants (Basel). 2021 Sep 28;10(10):2043. doi: 10.3390/plants10102043 (PMC8538535; doi:10.3390/plants10102043)
Supplement: Supplementary file 1 [file plants-10-02043-s001.zip › Table S1.pdf]

Table S1. Normalized transcription levels of *αCENH3* and *βCENH3* in different tissues of rye (*S. cereale*)

Tissue sample	'Imperial'			'Korotkostebel'naya 69'		
	αCENH3*	βCENH3*	αCENH3/ βCENH3	αCENH3*	βCENH3*	αCENH3/ βCENH3
Radicle	0.41 ± 0.01	0.14 ± 0.02	2.9	0.42 ± 0.02	0.22 ± 0.04	1.9
Coleoptile	0.82 ± 0.02	0.18 ± 0.01	4.7	0.66 ± 0.04	0.22 ± 0.03	3.0
Leaf	0.12 ± 0.02	0.06 ± 0.04	2.0	0.22 ± 0.05	0.10 ± 0.08	2.2
Stem	0.14 ± 0.07	0.32 ± 0.09	0.4	0.06 ± 0.03	0.19 ± 0.04	0.3
Anther	1.71 ± 0.08	0.97 ± 0.03	1.8	1.89 ± 0.29	1.32 ± 0.06	1.4
Carpel	3.64 ± 0.25	1.77 ± 0.21	2.1	3.88 ± 0.20	3.16 ± 0.35	1.2

The transcription levels of the *αCENH3* and *βCENH3* genes in rye were normalized using two reference genes, Ta2776 (RNase L inhibitor-like protein) and Ta53967 (Vacuolar ATP synthase 16 kDa proteolipid subunit). * Means of target/reference ratios. Standard Deviation (SD) was calculated with the STDEV function, Microsoft Excel 2007.
