# Supplementary material for: Expression of Two Rye CENH3 Variants and Their Loading into Centromeres
Source: Plants (Basel). 2021 Sep 28;10(10):2043. doi: 10.3390/plants10102043 (PMC8538535; doi:10.3390/plants10102043)
Supplement: Supplementary file 1 [file plants-10-02043-s001.zip › Table S2.pdf]

Table S2. List of primers used in this study

Primers	5' - 3' Sequence
Ta2776 F	CGATTCAGAGCAGCGTATTGTTG
Ta2776 R	AGTTGGTCGGGTCTCTTCTAAATG
Ta53967 F	GCTGGAGTCAGGGCTAAT
Ta53967 R	CGACAATGAGACCGTAGAGA
αCENH3 F	GCACCATTTGTCCGTCTGAT
αCENH3 R	GATACTCTGCAGCCTCTTGAAT
βCENH3 F	TCGTCCGCCTGGTTAAGGA
βCENH3 R	TGATACTCTGCAGCCTCTTGC
